# Using the Delphi technique to determine objectives and topical outline for a pharmaceutical care course: an experience from the Cuban higher education system

**DOI:** 10.1186/s12909-021-02583-1

**Published:** 2021-03-16

**Authors:** Alina M. Martínez-Sánchez

**Affiliations:** 1 Facultad de Formación de Profesorado y Educación, Universidad Autónoma de Madrid, Ciudad Universitaria de Cantoblanco, c/ Francisco Tomás y Valiente, 3, 28049 Madrid, Spain; 2 Ex - Pharmacy Department Director, Universidad de Oriente, Santiago de Cuba, Cuba

**Keywords:** Delphi questionnaire, Pharmacy education, Pharmaceutical care

## Abstract

**Background:**

Being pharmaceutical care one of the four areas defined by the International Pharmaceutical Federation’s Global Competence Framework, the curriculum redesigned scheme is a priority task to perform a pharmaceutical workforce capable to contribute significantly to the appropriate use of medicines. Therefore, the pharmacy curriculum should be adapted, in order to provide pharmacists with new knowledge and skills to provide pharmaceutical care services. This study used a modified Delphi technique to define objectives and topical outlines for a pharmaceutical care course on a pharmacy curriculum.

**Methods:**

A modified Delphi process was used to determine a consensus among proposed course objectives and topical outlines. The preliminary phase of the study included a compilation of prospective objectives and outline topics on which to structure informational flow through the Delphi. A two-round modified Delphi process were completed by the participants in the study. The Delphi questionnaire was organized using six domains: the theoretical program foundation; recommended for teaching literature; instructional and educational objectives of the program (course structure); teaching methods; knowledge, skills and professional values considered; and students’ performance assessments. Nineteen items for evaluation within the referred domains were considered.

**Results:**

Consensus was achieved among 15 participants regarding 10 objectives, and eleven topical outlines related to pharmaceutical care teaching in an undergraduate pharmacy course. Despite this favorable valuation and considering the qualitative evaluations provided by the participants, it was believed appropriate to analyze the recommendation for the inclusion of literature for the teaching of the course in Spanish language (73%). It resulted in a project proposal for the elaboration of a book by a group of authors from all the faculties of pharmacy in the country.

**Conclusion:**

A Delphi expert panel achieved consensus on topical outline and objectives for a pharmaceutical care course. The results of this study can be used to underline the didactic guidance for pharmaceutical care teaching and learning useful for future pharmacy curriculum upgrades.

**Supplementary Information:**

The online version contains supplementary material available at 10.1186/s12909-021-02583-1.

## Background

Since the concept of Pharmaceutical Care was announced from United States, this move has become an influential procedure of practice for many pharmacists around the World [1]. Pharmaceutical care is a professional practice that has arisen to meet the social need to prevent, identify, and solve drug therapy problems (DTP) and, therefore, reduce drug-related morbimortality [2, 3]. DTP is any unwanted incident related to medication therapy that actually or potentially affects the desired goals of treatment [4]. DTP is an important public health problem and has been heightened in recent years [5]. Relatively 28% of all emergency department visits and 5 to 10% of all hospital admissions are drug related [6, 7]. The estimated annual cost of drug-related morbidity and mortality resulting from nonoptimized medication therapy for the United States of America was $528.4 billion, equivalent to 16% of total US health care expenditures in 2016 [8]. Thus, DTPs represent a great challenge in the healthcare system as a result of increased patient morbidity, mortality, and healthcare costs [9]. In this stage, pharmaceutical care emerges as a patient-centered practice, in which the pharmacist assumes responsibility for the patients’ pharmacotherapeutic needs and collaborates with other members of the health-care team, becoming then co-responsible for the outcomes of their pharmacotherapy [10].

Pharmacy education around the world is witnessing a process of transformation leading to perform pharmacists capable to provide pharmaceutical care [11–13]. Profound curricular changes have occurred in countries like Australia, Chile and The United States of America, developing a patient centered care professional practice [14, 15]. But, even today, pharmacy education continues to be a barrier for the implementation and development of the pharmaceutical care [16]. As long as, how to implement the pharmaceutical care in a pharmacy curriculum is an unanswered question in some countries around the world. In addition, pharmacy students have difficulties with the practice of pharmaceutical care, despite having extensive knowledge on medicines, especially in developing countries [17, 18].

The pharmacy curriculum should be adapted, in order to provide pharmacists with new knowledge and skills. In most countries this change is taking place but not in a very structured manner [19]. Fresco and Silva described an example of pharmaceutical care teaching experience showing a description of a course syllabus, methods and teaching evaluation tools employed that demonstrated to motivate students toward this new practice and prepare them to increase their practical skills and clinical competences [20]. Furthermore, Fernández-Llimos and Nunes da Cunha designed a coding tree to classify pharmacy practice teaching topics, they included 110 pharmacy schools with 1703 pharmacy practice syllabus related to the pharmacy practice area, nonetheless, according to the authors, other studies are necessary to note the teaching of these curricular topics [21]. Recently, Arason et al., refers this type of learning more challenging than just learning content to repeat or apply at a later time. It is necessary to personalize this learning and make the practice of pharmaceutical care “real” for students. How do we facilitate the incorporation of Pharmaceutical Care and Pharmacists Patients` Care Process (PPCP) into our students’ professional identity development was a question stated by the same author, and the proposal method is to introduce the PPCP early in the curriculum and link it to aspects that the profession students are familiar with, similarly, this approach was described by Martinez-Sanchez in 2009 [22, 23].

Being the pharmaceutical care one of the four areas defined by the International Pharmaceutical Federation’s Global Competence Framework, the curriculum redesigned scheme is a priority task to perform a pharmaceutical workforce capable to contribute significantly to the appropriate use of medicines through research and by active participation in pharmaceutical care in collaboration with patients and other health care professionals [24, 25].

In 2005, the Cuban Government adopted inventiveness to inspire pharmacists to apply this new professional practice, including pharmaceutical care in the Cuban pharmacy legislation. Recommendations for didactic and assessment chances concerning incorporation of pharmaceutical care into the pharmacy curriculum, and the foundation for this emerging area of curriculum were included. In 2017, the Cuban Council on Higher Education stated a new pharmacy curriculum, consisting on pharmaceutical care to be considered as an eligible topic into the framework of a self-obligatory basic curriculum according to the necessities related to the social and geographical context university [26]. Nevertheless, limited published guidance is available on curricular structures, determining curricular objectives, topics and skills for pharmaceutical care in the Cuban pharmacy education system.

The Delphi Technique is a method designed to attain consensus of ideas of a group of experts through a series of intensive surveys interspersed with controlled belief feedback [27]. The basic argument of the Delphi research technique towards a pharmacy curriculum improvement, is entrenched in some form of general agreement and consensus regarding the core ingredients and components of the subsequent framework. Given the current status of implementing pharmaceutical care practice and education in developing countries, and the absence of generally based guidelines, the search for consensus and a point of departure in issues on clinical pharmacy education that will better serve developing countries is therefore justified through the use of this technique [28, 29]. Many studies support their developing and informing curriculum, thus, this technique have been found useful in curriculum development in business, nursing, medicine, agriculture, and technology along with environmental education [30]. The aim of this modified Delphi study was to determine objectives and topical outlines for a pharmaceutical care course on a pharmacy curriculum.

## Method

This study used a modified Delphi technique to obtain consensus for undergraduate pharmaceutical care course determination of objectives and topic outlines on a pharmacy curriculum. The study site was the University of Oriente Pharmacy Department in Santiago of Cuba (Cuba).

### Delphi expert panel

Pharmacy professors from seven national and foreign pharmacy schools and pharmaceutical organizations were invited to take part in this study. Participants from United State of America, Chile, Spain and United Kingdom were considered due to the recognized experiences of these countries in clinical and pharmaceutical care education and practice. The aim of the study was explained in the invitation letter. Consent for participation in the study was obtained by e-mail.

A call for participants was sent via email to members of the International Pharmaceutical Federation (FIP), American Association of Clinical Pharmacy (AACP), European Society of Clinical Pharmacy (ESCP), Iberian and Latin American Pharmaceutical Organization (Spanish OFIL), and the Cuban Pharmacy Association. We selected 15 pharmacy professors who had a background from at least three of the following criteria (some represented more than a few criteria): 1) Accreditation Council for Pharmacy Education membership. 2) Chaired or led a study or project of a school’s PharmD and/or Pharm. BSc program, 3) Served as an officer for a pharmacy education evaluation-related special interest group (SIG) within a professional organization, 4) Have authored a peer-reviewed publication focused on clinical pharmacy competence education. The establishment of the criteria was led by the literature [31–35]. Teachers who are not PharmD and/or are not involved in clinical pharmacy teaching were excluded. Expert coefficient competence ‘K’ was calculated, which is considered a rate of the level of competence in a constituent panel of experts. In this study greater than 0.8 was considered high and for the group of coefficient ‘k’ low was less than 0.7 [36].

In Cuba, pharmacy students undergo five years of undergraduate training before graduating as BSc. Pharm. Cuban pharmaceutical programs include both basic education on chemical, physiology and biological sciences and pharmaceutical practice at the community and hospital settings. In addition, to funding a clinical pharmacy training disciplines like Anatomy and systems-based pharmacology and therapeutics are included in a modularized and integrated curriculum. To perform a pharmacist capable to provide pharmaceutical patient- centered services a Social Pharmacy discipline was introduced in the curriculum. Enquiries concerning drug information, patient counselling, drug-related problems, and compliance are analyzed offering a 120-h internship to pharmacy practice [37, 38]. In short, a pharmacy student receives formal education about clinical pharmacy and pharmaceutical care during the third and fourth year and clinical pharmacy training (usually from the fourth to the fifth year). Even though there is not a Cuban guideline on which undergraduate pharmaceutical care education or training is based. However, each academic institution chooses their own curriculum and appoint subjects related to clinical pharmacy and pharmaceutical care education according to its geographical health necessities [39]. After graduation pharmacists are registered as public practitioners and appointed to work at the public services as community or hospital pharmacists for there is no private opportunity in the Cuban Health System. Few opportunities to work in the pharmaceutical industry happen. All pharmacists are compelled to fulfil a social service during the first two years. After, they have some opportunities to be eligible to postgraduate programs such as Master or Doctor in Pharmaceutical Sciences; pharmaceutical internship programs are nonexistent.

### Delphi survey development

The preliminary phase of the study included a compilation of prospective objectives and outline topics on which to structure informational flow through the Delphi. A bibliography examination was conducted on three pharmacy education-related journals: the *American Journal of Pharmaceutical Education*, *Pharmacy Education*, and *Journal of Pharmacy Practice and Education*. All journals were queried using the examination terms “pharmaceutical care”, “pharmacy education” and “curriculum” to categorize potential issues of interest. In addition, the books entitled *“Pharmaceutical Care Practice” and “Pharmaceutical Care Practice: The Clinician’s Guide”* by Cipolle, R., Strand, L., Morley, P. were reviewed [40, 41]. These publications were selected for the organizational and curricular approach because it provided guidance on pharmaceutical care syllabus for pharmacists theoretical and practical training. The search was limited to English or Spanish written articles issued from September 1st, 2001 to January 1st, 2017. We selected the year 2001 as the starting line of the search time because the Cuban Pharmacy curriculum named “Program C1” was initiated at the beginning of 2001, for topics related to clinical pharmacy and pharmaceutical care practice were officially included in Cuban pharmacy education.

Course objectives and topics were obtained from the pertinent literature. Moreover, researcher members from the “Manuel F. Gran” Center for the Study of Higher Education at the University of Oriente in Santiago de Cuba, Cuba were informally invited to contribute course objectives/topic from their pedagogical approach. This was done to enhance contributions from a didactic point of view toward ensuring consistency in terms of the teaching relationships established between objectives, subjects and methods as didactical categories within curricular design processes. A final list of objectives and topics were structured into three subjects with their respective proposals for teaching methods.

Accordingly, the Delphi questionnaire was organized using six domain structure adopted from the Cuban Ministry of Higher Education Guideline to design a teaching program, named: about the theoretical program foundation; recommended for teaching literature; instructional and educational objectives of the program (course structure); teaching methods; knowledge, skills and professional values considered; and students performance assessment. Nineteen items for evaluation within the referred domains were considered (Additional file 1).

Potential pharmacy professors who could serve as consultants in a pilot questionnaire evaluation were identified using National Commission for Pharmacy Education membership listservs obtained from the Vocational Training Department at the Ministry of Higher Education. Furthermore, potential pharmacists were identified from the professional pharmacist’ register from Santiago de Cuba city. The questionnaire developed was reviewed by the authors for topic and layout and then piloted with two pharmacology professors from Havana University, and two community pharmacists developing clinical pharmacy activities from Santiago of Cuba, who were not included in the subsequent study panel. For the pilot study the questionnaire explaining purpose and requesting opinions related to understanding of pharmaceutical and pedagogical terms as well as clarity in questions structuring was delivered via email. This resulted in the rewording of a `pharmacist with a wide profile´; this Cuban pedagogical definition according to which a professional profile is linked to a broad basic training [42]. Other modifications were not required.

### Delphi survey process

The Delphi technique was used to attain a consensus on the objectives and topic outline for a pharmaceutical care course in a pharmacy curriculum. The Delphi method is a consensus-based technique that offers a systematic method of collecting and aggregating informed judgments from a group of experts via several iterations. Controlled feedback from successive rounds encourages participants to reassess, alter and/or develop opinions [43]. The Delphi process depends on the identification and use of specialists within the subject area. Delphi process does not require a random sample and that the qualities of the panel members are more significant than the number of persons on the panel.

The Delphi technique has been applied widely in diverse areas of scientific research [44]. Similarly, Delphi studies have been valuable in educational settings in forming procedures, standards, and in predicting tendencies [45]. In addition, numerous studies endorse the use of Delphi technique in pharmacy curriculum foundation [46–48].

A two-round modified Delphi process was completed by the participants in the study. We provided a base of objectives and topics recovered from the literature. In addition, a proposal of basic teaching literature was sent. Invitations were sent via e-mail, with the explanation of the study and the assurance of their participation anonymity.

The questionnaire was divided into two sections. Rounds included a description of the targets and approval for participation and instructions for evaluation. The first section registered demographic information: age, gender, professional qualification, years of work experience in pharmacy, teaching experience in clinical pharmacy and/or pharmaceutical care. Objectives and topics were assessed across each of the questionnaire domains and its items using the 5-point Likert scale indicating strength of importance for each objective (5 = extremely suitable, 4 = very suitable, 3 = moderately suitable, 2 = slightly suitable, and 1 = not at all suitable). For the first round, respondents were given a chance to suggest modifications to current objectives/topics within each domain for evaluation.

Responses to the questions asked in the first iteration were synthesized and used for the second round. Only the items or points where consensus was not reached in the first stage were included in this round. The participants were asked to reconsider their scores having studied the whole panel’s anonymized responses. They were provided with the following: (1) median and interquartile range of the whole panel’s response for each definition or scenario; (2) comments made by individual (anonymous) participants together with the associated score; and (3) their own score relating to an item or definition. The inclusion of the participants’ comments and a summary of their responses increase the number of reasoned responses and decreases the number of rounds required in order to reach consensus.

### Data analysis

Statistical data was analyzed using the automated system “Delphi”, on a work platform in Excel for Windows developed by Gómez et al. [49]. Descriptive statistics were used to illustrate respondents’ demographic characteristics and group responses to each domain in all two rounds. According to Aronson and colleagues, there is a large number of definitions for the desired level of consensus in a Delphi process, but there is not an ultimate agreement [50].

In our case, with the dual objective of evaluating the appropriateness of the direct assignation of values to the responses of the ordinal scale in the evaluation of the items of the questionnaire by the judges participating in the Panel of Experts for their quantification, and analyze the suitability of the items raised in the Delphi method for the composition of the questionnaire the following steps are developed:

1. A summary table of the various weightings provided by the expert judges was drawn up assigning an assessment of the responses according to the criterion:
2. From the assessments, absolute response frequencies and accumulated frequencies were calculated, as well as the relative cumulative frequency, obtained from the quotient between the cumulative frequency of responses and the number of existing responses or number of experts, the latter expressed to two decimal places. It is interesting to observe how the relative accumulated frequency saturates its maximum value before the first category accumulating its maximum probability in all questions, so that the minimum indicator present will be “inadequate”.
3. The cumulative relative frequencies were used to calculate the cut-off points and their respective indicator scales using the inverse standard normal values of each indicator’s own cumulative probabilities in each question. To do this, we used the approximation to the nearest value of the Standard Normal curve of cumulative probability. It is necessary to indicate that for cumulative probability values equal to 1, the corresponding inverse standard value is considered 3.5 as a practical reduction when asymptotic from 3.49. Similarly, for cumulative probability values equal to 0, the inverse standard value shall be assumed to be equal to − 3.5. Having taken this into account, and in order to facilitate the work of calculating the inverse standard function corresponding to each cumulative probability value, the function relative to the inverse of the standard function was made use of through a computer spreadsheet software.
4. The “Average” column was added to the calculated values, obtained from the calculation of the average of the values found per row. Similarly, Cutoff Points are calculated as the average of the values of the inverse standard function for each of the scale values (columns). The value of Limit N was also determined, through the average of the Cut Off Points (whose result will be the same as the average of the averages of each category) and that will delimit the true ranges of interval to which pertains each category.

To determine the actual belonging said the ranges of each category for each item the value N-P was estimated, obtained as the difference of the limit value less the average value of each item. Finally, by comparing the N-*P* value of each item with the cut-off points and range limits of each of the categories, the belonging of each of the items is precisely determined [51].

## Results

Characteristics of responders participating in the panel process are shown in Table 1. Of the 15 respondents, 14 were male (93%) and 1 was female (6%). Respondents were also experienced in pharmacy teaching and practice (average of 20 and 15 years of professional experience and clinical pharmacy teaching experience, respectively). All experts were PharmD. Most of respondents presented 86% of the experts presented a high competence coefficient, 24% presented a medium competence coefficient (Table 2). The presented information of the coefficient ‘k’ to the experts of the Delphi study concerning objectives and topic online for a pharmaceutical care course in a pharmacy curriculum was obtained from the information intreated from the participants, about their self-worth in terms of degree of knowledge on the theme and level of argumentation.

**Table 1:** Characteristics of respondents participating in panel

Respondents	Age	Gender	Professional qualification	Work experience in pharmacy	Teaching experience in clinical pharmacy and/or pharmaceutical care	Country
1	54	Female	PharmD	21	14	*Cuba*
2	60	Male	PharmD	20	15	*Cuba*
3	65	Male	PharmD	18	11	*Cuba*
4	55	Male	PharmD	22	15	*Cuba*
5	49	Male	PharmD	20	20	*Cuba*
6	57	Male	PharmD	16	12	*Cuba*
7	60	Male	PharmD	19	13	*Cuba*
8	54	Male	PharmD	20	14	*Cuba*
9	55	Male	PharmD	21	14	*Cuba*
10	59	Male	PharmD	23	19	*EEUU*
11	62	Male	PharmD	20	17	EEUU
12	58	Male	PharmD	26	10	Chile
13	60	Male	PharmD	27	17	Spain
14	52	Male	PharmD	17	15	United Kingdom
15	58	Male	PharmD	23	20	United Kingdom

**Table 2:** Expert competence coefficient “k”

Participants	Kc	Ka	K	Proficiency Level	
1	0.6	0.8	0.7		Medium
2	1	1	1	High	
3	0.9	1	0.9	High	
4	0.9	1	0.9		Medium
5	0.7	0.8	0.7	High	
6	0.8	0.9	0.8	High	
7	0.7	1	0.8	High	
8	0.9	0.9	0.9	High	
9	0.9	0.9	0.9	High	
10	0.8	0.9	0.8	High	
11	1	0.9	1	High	
12	0.8	1	0.8	High	
13	1	1	1	High	
14	0.7	0.8	0.7		Medium
15	0.9	1	0.9	High	

*K* Expert competence coefficient (scale of 0.25 to1)

*Ka* ‘coefficient of argument’ or foundation of expert opinion. It is obtained from assigning scores to a number of different sources of argument that could wield the expert

*Kc* ‘coefficient of knowledge’ or information that the expert on the subject or problem posed. It is calculated from the assessment made by the expert himself on a scale of 0 to 10, multiplied by 0,1

**K = ½ (Kc + Ka)**

### Pharmaceutical care course

After literature review was possible to identify three main steps of the pharmaceutical care process: patient’s therapeutic necessities evaluation; design and establishment of a care plan and the evaluation of therapeutic goals. Three main subjects based on these steps were defined as follows: Integrated Pharmaceutical Care I, II and III. These subjects comprised the pharmaceutical care course designed. Issues such as: 1) understanding Pharmaceutical Care as a philosophy of practice; 2) pharmaceutical care process has a logical expression, in the clinical pharmacy method; 3) the clinical pharmacy method results to apply, the scientific method in the patient-drug relation managing; 4) the scopes of knowledge to curricular structure should be determined by patient needs and 5) the teaching process must consider the values inherent to the practice of pharmaceutical care were considered foundations to the pharmaceutical care course outline. For the most part of the respondents the theory and practical foundation course was considered very suitable (100%).

### Course objectives and topical outline

Literature review resulted in 15 possible objectives. The list was refined to 12 objectives after categorization and duplicate removal. These 12 objectives were tested in the first round. Two objectives were eliminated after detecting duplication in terms of skills according to Bloom’s taxonomy. From a pedagogical point of view, two objectives were eliminated after detecting duplication in terms of skills according to Bloom’s taxonomy. From a pedagogical point of view, these objectives included the same abilities and knowledge than other objectives considered in the final list. This list was refined to 10 objectives (16% reduction) after the first round. Over the second round, nor change was proposed. Accordingly, the final accepted list (after the second round) consisted of 10 objectives distributed according to the skills and content corresponding to each of the three subjects described for the course. The Delphi process resulted in consensus, with 100% expert agreement with the topical outline after the completion of the first round. Eleven topical outlines were related to course objectives including principles of learning by emphasis on patient experiences related to medication, drugs and disease states and technical knowledge of how to provide pharmaceutical care practice, evaluation of drug - related problems and communications (Additional file 1). Topical outlines ranked suitable or very suitable were similarly associated to communication with physicians and clinical knowledge, developing an ethic framework, health-enhancing behaviors, avoid or reduce health risks associated to drug use.

After the second round, consensus was reached for all items in the questionnaire (Table 3). After the distribution in the different categories, it is observed that the evaluations provided by the members of the Panel of Experts are highly favorable to the items proposed in the questionnaire (Table 4).

**Table 3:** Determination of images by the Inverse Standard Normal Curve and the Cutoff Points

Items	Very suitable	Suitable	Unsuitable	Averages	N-P
**About the theoretical and practical foundation**					
Q1	0.84	3.49	3.49	2.60	−0.621
Q2	3.49	3.49	3.49	3.49	−1.511
Q3	−0,23	3.49	3.49	2.25	− 0.271
**Required and optional literature**					
Q4	1.48	3.49	3.49	2.82	−0.841
Q5	−0.10	1.48	3.49	1.62	0.359
Q6	1.48	3.49	3.49	2.82	−0.841
Instructional and educational objectives					
Q7	3.49	3.49	3.49	3.49	−1.511
Q8	1.48	3.49	3.49	2.82	−0.841
**Course structure**					
Q9	0.25	3.49	3.49	2.41	−0.431
Q10	−1.13	3.49	3.49	1.95	0.029
Q11	−0.43	3.49	3.49	2.18	−0.201
Q12	−1.55	3.49	3.49	1.81	0.169
**Content**					
Q13	3.49	3.49	3.49	3.49	−1.511
Q14	3.49	3.49	3.49	3.49	−1.511
Q15	0.62	3.49	3.49	2.53	−0.551
**Teaching methods**					
Q16	0.84	3.49	3.49	2.60	−0.621
Q17	0.25	3.49	3.49	2.41	−0.431
**Evaluation**					
Q18	1.11	3.49	3.49	2.69	−0.711
**Cut points**	**1.048**	**3.378**	**3.49**		

Decision rule:

Very suitable - 1048; Suitable - 3378; Not very suitable - 3,49

If N - P < Cut-off point, then it is accepted as valid within the corresponding category

N: is the result of dividing the sum of the sums by the product of the number of categories by the number of steps

P: averages

N - P: Consulted experts given average value to each item

**Table 4:** Rank belonging to each of the items

Items	N-P	Category
Q1	−0.621	Very suitable
Q2	−1.511	Very suitable
Q3	−0.271	Very suitable
Q4	−0.841	Very suitable
Q5	0.359	Very suitable
Q6	−0.841	Very suitable
Q7	−1.511	Very suitable
Q8	−0.841	Very suitable
Q9	−0.431	Very suitable
Q10	0.029	Very suitable
Q11	−0.201	Very suitable
Q12	0.169	Very suitable
Q13	−1.511	Very suitable
Q14	−1.511	Very suitable
Q15	−0.551	Very suitable
Q16	−0.621	Very suitable
Q17	−0.431	Very suitable
Q18	−0.711	Very suitable

Despite this favorable valuation and considering the qualitative evaluations provided by the participants, it was believed opportune to analyze the recommendation for the inclusion of literature for the teaching of the course in Spanish (73%). It resulted in a project proposal for the elaboration of a book by a group of authors from all the faculties of pharmacy in the country.

## Discussion

This modified Delphi study was a first effort to achieve consensus on a pharmaceutical care course in a curriculum for pharmacy students. The changeable training opportunities offered by pharmacy faculties and the lack of national guideline for an undergraduate pharmaceutical care curriculum was a motivation for performing this study. Every discussion around the Pharmacy education programs must begin with an interpretation of the profession’s purpose within society. Societies funding favored status to professions because their members possess specialized knowledge. Intrinsic in this is a pact in which professionals use their expert capabilities in the best concern of the public. Consistent with Cuban economic and social reality the mission of the pharmacy profession is to deliver drugs to people with growing health care demands, and to help citizens achieve the best effects from pharmacotherapies, therefore enhancing health system [52]. The theoretical input of this study lies in submitting a syllabus from a didactical perspective to implement pharmaceutical care on a pharmacy curriculum, considering topics and skills as a system.

On the other hand, the validation of the objectives of this program through the Delphi method comes to demonstrate the validity of the didactic interpretation of professional processes and their translation in the process of curriculum design, being another contribution of this study. The most important feature is to recognize pharmaceutical care as a philosophy. Pharmaceutical care is a generalist practice that can be applied in all setting: community, hospital, long-term care, and the clinic. It can be used to care for all types of patients with all types of diseases taking any type of drug therapy [53]. Since a pedagogical point of view: it is a professional way of performance. Subsequently, is not possible to include pharmaceutical care in the curriculum, merely altering name matter related clinical pharmacy by pharmaceutical care.

Through an iterative process of thoroughly assessing agreement amongst clinical and pharmaceutical care experts, 10 objectives reached consensus to be included in pharmaceutical care course designed. There was also considerable agreement on certain skills and abilities, regardless of the practice setting. All respondents were in agreement that designing individualized, culturally and therapeutic care plan, resolving drug-related problems, communication with health care professionals, implement a therapeutic plan, integration knowledge, skills, and personal caring into the provision and process of pharmaceutical care were very suitable. From the system perspective applied to the teaching and learning process it is not possible to separate the analysis and design of the objectives from the content established for teaching. Across the process, the clinical pharmacy method is applied, and the dialectic relation problem- method is showed. Consequently, knowledge, skills and professional values are included in this relation, and is feasible to determinate the highly aspect to transform into the teaching process; the method comes to be part of this content [54]. In line with pharmaceutical care practice the pharmacy student needs to be training to design, implement and monitoring a therapeutic plan to identify, resolve, and prevent drug related problems [55]. Therefore, these skills have been considered in the core of the objectives established for teaching pharmaceutical care.

For the student to appropriate a patient-centered performance logic the curriculum requires a sequence of objectives, which specify the expected outcomes of a pharmaceutical care process at each level. In this study, the process of curricular design of objectives and topical outline was dominated by the logic of the pharmaceutical care process taking into consideration each of its stages, starting with the evaluation of the patient’s needs until the evaluation of its therapeutic results based on the improvement of the patient’s quality of life. Identifying topical outlines support the professors in designing the framework, covering a course objective. Consequently, the topical outlines were designed and revised to support the course objectives. The surveyed participants showed their satisfaction with the topical outlines attached to course objectives like pharmaceutical care; introduction & impact care planning as a component of the patient care process; drug therapy problems and tools for evaluating the quality of care offered. Consensus can be explained by the fact that respondents have a high experience level in clinical pharmacy practice and teaching, similarly, in the pharmaceutical care area there is some uniform worldwide theoretical framework around. In this regard, because items like inadequate clinical knowledge, and communication skills of pharmacists have been described as barriers have hampered the implementation of pharmaceutical care was an imperative design a broad and encompassing topical outline [56, 57]. Consistently, drug-related problems were widely considered in the topical outline. Being the core of the pharmaceutical care process scientific literature considers its inclusion, relevant in a pharmacy curriculum [58].

It has also been described that pharmacists, as givers to patient care, should evaluate data concerning untoward effects of drugs and be well competent to recognize and prevent these drug-related problems [59, 60]. The pharmaceutical care concept accepts clinical interventions which lead to most favorable health outcomes. Identification, prevention or resolution of DTPs enhance patient’s health results, and consequently, it should be incorporated within pharmaceutical care [61]. Thus, knowledge and training are essential requirements to competently deliver pharmaceutical care [62].

Using this curriculum framework, the pharmacy professor may now put these into operation for their specific subjects using existing resources, such as Hospital and Community position policies and papers. This study also confirmed other suggestion of our respondents for including some Spanish language bibliography in pharmaceutical care teaching. The findings of the study could facilitate students to have firm foundation of pharmaceutical care required for pharmacy practice, which they can build upon during their future training. It can help Social Pharmacy teachers to have specific outcomes which are clinically relevant in current clinical pharmacy practice.

The proposal to develop a basic book for the teaching of pharmaceutical care by a group of authors from all faculties of pharmacy is a challenging idea and at the same time an excellent opportunity to contextualize the teaching and practice of pharmaceutical care in the country. Since pharmaceutical care is an internationally accepted philosophy of professional practice, it is important that in each implementation strategy the particularities of the educational and health systems of each country are taken into consideration. Meanwhile, this new model will require profound transformations in terms of teaching methods, resources and the establishment of new forms of university-industry relations. Likewise, health systems will have to reform their models in terms of structures, processes and results to make room for a new professional who will be inserted with a concrete process to contribute to public health. All this knowledge, skills and attitudes should be taught in pharmacy schools and reinforced in clinical practice settings.

In addition, this methodology succeeds in deriving the key elements with regards to objectives and knowledge concerning pharmaceutical care process, besides it guides us about the pedagogical approach needed to design these elements in a pharmaceutical care teaching program. The objectives and topical outline identified in this study are dynamic and are possible to vary over time with progress of pharmaceutical care practice and may need to be reviewed in a near future.

Asking and answering fundamental questions required to define and validate curriculum design elements contained in a Pharmaceutical Care teaching program. Although defining and describing these elements were necessary steps toward developing a theoretical model to implement pharmaceutical care in pharmacy curriculum, additional questions remain and will need to be undertaken with future research. The inclusion criteria were another limitation of this study. Although they aimed to gather a group of individuals who were pharmacy education experts, our need for objective criteria that was available in accessible records may have resulted in the exclusion of individuals who had significant experience in pharmacy teaching.

## Conclusion

This study provides the first evidence for pharmacy education curriculum modification toward pharmaceutical care teaching undergraduates based on the expert panel. This study has helped standard pharmacy practice knowledge requirements that are most relevant to present pharmaceutical care teaching, and essential in teaching pharmacy students. It can be used to underline the clinical significance for pharmaceutical care teaching and learning useful for future both, clinical practice and pharmacy education. Thus, it can facilitate students to gain a better understanding of how pharmaceutical care knowledge is operated in clinical practice. The results of this study can be used to underline the didactic guidance for pharmaceutical care teaching and learning useful for future pharmacy curriculum upgrades.

## Supplementary Information

**Additional file 1.** Appendix 1. Definition of domains and items used in the questionnaire layout. Appendix 2. Final list consensus objectives related to topical outlines for undergraduate in pharmaceutical course.

## Data Availability

All data generated or analyzed during this research are included in this article.
