# Supplementary material for: Using the Delphi technique to determine objectives and topical outline for a pharmaceutical care course: an experience from the Cuban higher education system
Source: BMC Med Educ. 2021 Mar 16;21:158. doi: 10.1186/s12909-021-02583-1 (PMC7962236; doi:10.1186/s12909-021-02583-1)
Supplement: Supplementary file 1 — Additional file 1. Appendix 1. Definition of domains and items used in the questionnaire layout. Appendix 2. Final list consensus objectives related to topical outlines for undergraduate in pharmaceutical course. [file 12909_2021_2583_MOESM1_ESM.docx]

**Appendix 1. Definition of domains and items used in the questionnaire layout.**

| **Domains** | **Definition of contents for items** |
| --- | --- |
| About the theoretical and practical foundations | \| Relevance in relation to the professional model (Professional Profile); \| \| --- \| \| Relationship with the current demands of the pharmaceutical profession; Didactic foundation \| \|  \| |
| Required and optional literature | \|  \| \| --- \| \| Origin and language diversity; Correspondence with the objectives \| \|  \| \| Instructional and educational objectives; Clear and precise design; Relevance \| \|  \| \|  \| |
| Course structure | \| Correspondence with the objectives; Feasibility of practical activities; Sequence; Distribution and  balance of schedule \| \| --- \| \|  \| \|  \| \|  \| |
| Content | \| Relevance; Correspondence with the objectives; Systematicity \| \| --- \| \|  \| \|  \| |
| Teaching methods | \| Student responsibility for education; Problem basic approach \| \| --- \| \|  \| |
| Evaluation | Assess application to practice |

**Appendix 2. Final list consensus objectives related to topical outlines for undergraduate in pharmaceutical course**

| **Subjects** | **Objectives** | **Topics** |
| --- | --- | --- |
| Integrated Pharmaceutical Care I | - Design individualized, culturally and clinically appropriate care plans. - Interviewing a patient. - Completing a clinical history - Completing drug utilization - Identify drug therapy problems. | [Pharmaceutical Care – Introduction & Impact](http://faculty.ksu.edu.sa/hussain/Documents/PHCL%20429%20Introduction%20and%20Impact%20Fall%202010%20BW%20HO.pdf)  [Care planning: a component of the patient care process](http://faculty.ksu.edu.sa/hussain/Documents/CARE%20PLANNING%20A%20COMPONENT%20OF%20THE%20PATIENT%20CARE%20PROCESS.pdf)  [Drug Therapy Problems](http://faculty.ksu.edu.sa/hussain/Documents/PCHL%20429%20%20DRP%20Fall%202010%20BW%20HO.pdf)  [Subjective and Objective Information](http://faculty.ksu.edu.sa/hussain/Documents/PCHL%20429%20%20Subjective%20Objective%20Information%20Fall%202010%20BW%20HO.pdf)  [Care Plan, Follow-up and Evaluations](http://faculty.ksu.edu.sa/hussain/Documents/plan,%20evaluation,%20presnt%20%5bCompatibility%20Mode%5d.pdf) |
| Integrated Pharmaceutical Care II | - Implementing therapeutic plan - Advice patients about drug choices and other treatment options - Address patient concerns / resistance / ambivalence and cultural consideration - Document pharmaceutical care activities for ongoing patients care quality control, quality assurance, and accountability. | Levels of pharmaceutical care: factors and risks  Pharmaceutical interventions more common in clinical practice.  Communication skills in pharmacy practice |
| Integrated Pharmaceutical Care III | - Integrate their knowledge, skills, and personal caring into the provision and process of pharmaceutical care | Tools for assessing the quality of care provided. Evaluation of pharmaceutical care process: model structure - process - outcome.  Methods to achieve quality of life. |
